# The Roles of CCN1/CYR61 in Pulmonary Diseases

**DOI:** 10.3390/ijms21217810

**Published:** 2020-10-22

**Authors:** Yin Zhu, Sultan Almuntashiri, Yohan Han, Xiaoyun Wang, Payaningal R. Somanath, Duo Zhang

**Affiliations:** 1 Clinical and Experimental Therapeutics, College of Pharmacy, University of Georgia and Charlie Norwood VA Medical Center, Augusta, GA 30912, USA; yinzhu@augusta.edu (Y.Z.); salmuntashiri@augusta.edu (S.A.); yohan@augusta.edu (Y.H.); sshenoy@augusta.edu (P.R.S.); 2 Center for Vaccines and Immunology, University of Georgia, Athens, GA 30602, USA; xiaoyunwang@uga.edu; 3 Department of Medicine, Augusta University, Augusta, GA 30912, USA

**Keywords:** lung injury, COPD, bronchopulmonary dysplasia, fibrosis, pulmonary hypertension, lung infection, lung cancer

## Abstract

CCN1 (cysteine-rich 61, connective tissue growth factor, and nephroblastoma-1), previously named CYR61 (cysteine-rich angiogenic inducer 61) belongs to the CCN family of matricellular proteins. CCN1 plays critical roles in the regulation of proliferation, differentiation, apoptosis, angiogenesis, and fibrosis. Recent studies have extensively characterized the important physiological and pathological roles of CCN1 in various tissues and organs. In this review, we summarize both basic and clinical aspects of CCN1 in pulmonary diseases, including acute lung injury (ALI), chronic obstructive pulmonary disease (COPD), lung fibrosis, pulmonary arterial hypertension (PAH), lung infection, and lung cancer. We also emphasize the important challenges for future investigations to better understand the CCN1 and its role in physiology and pathology, as well as the questions that need to be addressed for the therapeutic development of CCN1 antagonists in various lung diseases.

## 1. Introduction

**Figure 1:**
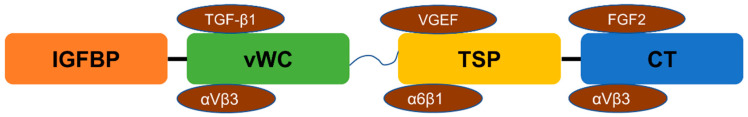
The structure of CCN protein and examples of their binding growth factors/integrins. The structure (from left to right) consists of insulin-like growth factor binding protein (IGFBP), von Willebrand factor type C repeat (vWC), thrombospondin type 1 repeat (TSP), and cysteine knot (CT). Transforming growth factor β1 (TGF-β1), fibroblast growth factor (FGF2), vascular endothelial growth factor (VEGF), and the integrins (αVβ5, α6β1, and αVβ3) are shown binding with each section.

The discovery of the CCN proteins started back in the 1990s, and many research groups have presented their work in regards to different CCN proteins [1]. The first found protein was named as the CCN1 or the CYR61, since the protein is recognized as a cysteine-rich protein. Then, CCN2/CTGF, CCN3/NOV, CCN4/WISP-1, CCN5/WISP-2, and CCN6/WISP-3 were introduced by researchers [1]. The CCN protein family consists of six secreted proteins with multifunction designated CCN1 to CCN6. These proteins are found to be conserved among different vertebrates, indicating their conserved roles during evolution. As shown in Figure 1, most of the CCN proteins contain a similar structure consisting of four functional domains: (a) insulin-like growth factor binding protein-like module (IGFBP); (b) von Willebrand factor type C repeat module (VWC); (c) thrombospondin type I repeat module (TSP-1); and (d) cysteine-knot-containing module (CT) [2]. One of the exceptions in the CCN protein family with respect to the structure is CCN5, which is lacking the cysteine-knot-containing module (CT), while other members share the same four modules [3]. In the four different modules, receptors such as integrin αVβ3, αVβ5, and α2Bβ3 will bind to the VWC module-binding site; integrin α6β1 will binding in the TSR module. In the CT module, many molecules like heparan sulfate proteoglycans (HSPG), integrin α6β1, αVβ3, α5β1, α6β1, and αMβ2 bind to the module [4,5]. These modules with the binding molecules provide multiple functions to the CCN proteins; like most of the matricellular proteins, CCN proteins can affect cellular behaviors based on different combinations of binding molecules. For example, integrins αvβ3 and heparan sulfate proteoglycans have several biological activities, including cell adhesion, migration, growth, apoptosis, and senescence [4,6,7,8,9]. When different integrins bind to the four modules, CCN proteins can function differently in various cell types.

Many in vivo studies, including animal and human trails, revealed that the CCN proteins might have the ability to enroll in the mediation process in human diseases. For example, the abnormal expression of the CCN proteins has been found in the diabetic nephropathy disease. The results have shown that the upregulated CCN2 is observed in the mesangial cells, as well as the decreasing expression of CCN1 in the podocytes in the diabetic retina [10,11,12]. In atherosclerosis diseases, the increased expressions of the CCN1 and CCN2 were also found where the inflammatory cells accumulated, practically around the plaques [13,14]. Moreover, the increased CCN1 level has been seen in rheumatoid arthritis disease. The researchers who reported these findings suggested a potential connection between the role of CCN1 protein and hyperplasia in the joint [15,16]. In the cancer category, CCN proteins have been extensively investigated, especially the dysregulation of CCN proteins in the development of several types of cancers. Based on the current findings, it has been observed that highly induced CCN1, CCN2, CCN3, and CCN5 were found in breast and ovarian cancer [17,18] along with the downregulation of CCN6 [19], which is correlated with poor prognosis situations. In lung cancer, there are many reports indicating that the CCN1 and CCN2 are negatively correlated with the prognosis of lung cancer, where the expression of CCN1 and CCN2 level is lower compared with normal matched lung tissues [20,21]. In contrast, there is a significant induction of CCN4 protein in the lung carcinoma samples [20]. In colon cancer, the increased CCN1, CCN2, and CCN4 were considered as an indicator for the tumor stages [22,23]. Based on this growing evidence, researchers suggested CCN protein members might serve as diagnostic markers and therapeutic targets in human diseases. As the investigations summarized above, the CCN protein family has shown promising potential for clinical usage. In this review, we focus on the CCN1 protein and its known roles in human lung diseases.

## 2. CCN1

CCN1 protein was first identified as a matricellular protein and was cloned from mouse 3T3 fibroblasts in the early 1990s [1]. Accumulated data suggested CCN1 is a multifunctional protein, which is essential for physiological processes such as embryonic development, tissue injury repair, senescence, and angiogenesis [24,25]. Later, CCN1 was also found to participate in pathological processes, including but not limited to arthritis, fibrosis, atherosclerosis, and cancer [26,27,28,29,30]. CCN1 is a rapid response gene to a wide range of stimulation. Many growth factors mediate transcriptional activation of CCN1, such as transforming growth factor β1 (TGF-β1), basic fibroblast growth factor (FGF2), growth hormone (GH), and platelet-derived growth factor (PDGF) [31]. Moreover, CCN1 also responds to stress stimuli, including hypoxia, hyperoxia, UV light, mechanical stretch, and bacterial as well as viral infections [32,33,34,35,36,37].

Proteolytic cleavage is an irreversible process in which the peptide bonds are broken by the proteases to acquire stable protein fragments [38], a process that also applies in the regulation of CC1 expression. Unlike the non-specific cleavage of the acid hydrolysis, a specific peptide bond is cleaved by the chosen protease, which only cleaves the particular amino acid [38]. Pendurthi et al. reported that plasmin, one of the protease families that has been involved in many cellular processes via the proteolytic cleavage to the extracellular matrix (ECM), has the potential ability to up-regulate the truncated form of CCN1 in breast carcinoma cells [39].

The expression of CCN1 has been found in multiple tissues, including lung, liver, heart, kidney, etc. [40,41,42,43]. Several cell types, such as epithelial cells, endothelial cells, neurons, and fibroblasts contribute to CCN1 expression in the tissues (Table 1). In adult human lungs, CCN1 is highly expressed in the pulmonary mesenchymal cell compared with epithelial, endothelial, or immune cells, according to the single-cell RNA-seq data generated by the LungMAP Consortium (www.lungmap.net, accessed on 21 September 2020).

The existing literature on CCN1 mainly focuses on its role in wound healing, angiogenesis, inflammation, and fibrosis, as well as on cancer research [9,24,54,58]. Integrins largely mediate the function of CCN1 via protein–protein interactions [59]. Binding with distinct integrins enables the diverse cellular functions of CCN1 [60]. For example, CCN1 interaction with integrin αvβ3 can promote cell survival, while binding with integrin α6β1 causes cell death [57,61,62]. On the other hand, CCN1 has been reported to interact with receptors other than integrins, such as VEGFR2, FGFR2, Notch, RANK, and TLR2/TLR4 [63,64,65]. The interaction between CCN1 and its receptors transduces signals to the downstream signaling partners that mediate the cellular functions (Table 2).

## 3. CCN1 in Lung Diseases

**Figure 2:**
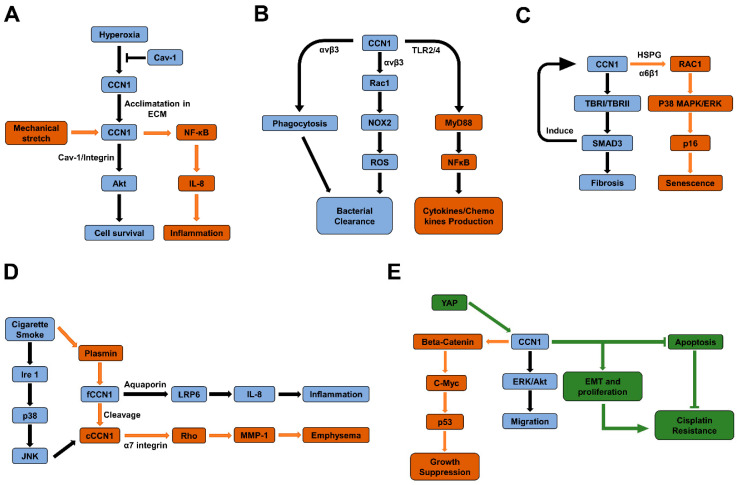
Schematic diagrams of signaling pathways mediate the pathogenesis of ALI in lung epithelial cells (**A**), pulmonary infection in macrophages (**B**), lung fibrosis in fibroblasts (**C**), COPD in lung epithelial cells (**D**), and lung cancer (**E**), respectively.

Studies have identified the involvement of CCN family members in the pathogenesis and disease progression to malignant and non-malignant lung diseases [20,44,73]. In the following sections, we present our current knowledge of CCN1 in various lung diseases and summarize the signaling pathways involved in their pathophysiology (Figure 2). The intention here is to encourage further investigations to characterize the in-depth molecular mechanisms to determine the clinical implication of CCN1 in https://www.webmd.com/lung/lung-diseases-overview (accessed on 21 September 2020) the pathogenesis of lung diseases with the long-term goal of targeting CCN1 and associated signaling pathways in developing therapeutics of lung anomalies.

### 3.1. CCN1 Association with Acute Lung Injury (ALI) and Bronchopulmonary Dysplasia (BPD)

Acute lung injury (ALI) and its severe form of acute respiratory distress syndrome (ARDS) cause significant morbidity and mortality worldwide [74,75]. A systemic review based on multiple databases showed that the mortality rate for ARDS was between 36.2% and 44.3% during the period 1984 to 2006 [76]. Despite decades of research on this topic, the pathogenesis remains unclear, and therapeutic strategies are still limited, which makes this devastating syndrome continue to be a major problem in intensive care units (ICUs).

In 2003, Perkowski et al. published a gene expression profile using the hyperoxic lung injury model in mice [77]. A significant increase of CCN1 was found in the lungs two days after hyperoxia exposure at the transcriptional level, suggesting its potential role in fibrosis, angiogenesis, and/or extracellular matrix remodeling [77]. Interestingly, in a hyperoxia-induced ALI experimental model in mice, it was not only CCN1 but also CCN2 expression that was observed to be upregulated in hyperoxic mouse lungs, which indicated that the CCN protein family plays a significant role in the pathogenesis of fibrosing alveolitis during the hyperoxic exposure of mice [78]. Furthermore, in a ventilator-induced lung injury (VILI) model in mice that was developed to mimic the lung-damaging effects of mechanical ventilation in acute lung injury patients, CCN1 expression was found to be upregulated significantly as a stress-activated gene [52]. In support of this observation, another study reported increased CCN1 mRNA expression in the mouse lung tissues subjected to VILI [79,80]. Similarly, in a bleomycin-induced experimental ALI in mice, CCN1 expression was found to be elevated in the alveolar mesenchymal cells, which in turn, activated the TGF-b1/SMAD3 pathway-dependent profibrotic signaling that contributed to ALI-mediated pulmonary fibrosis [81]. Overall, these findings provided substantial evidence to show CCN1 is actively involved in the pathogenesis of ALI.

Although the above reports demonstrate dysregulation of CCN1 in ALI/ARDS in several rodent models of ALI and fibroproliferation in the lungs, the precise role and the mechanisms by which CCN1 promotes injury and fibroproliferation in the lungs remains elusive. Studies from another group have provided some early insight into the role of CCN1 in hyperoxia-induced lung injury [70,82]. CCN1 was identified as a hyperoxia-induced protein in a variety of lung cells, including alveolar and bronchial epithelial cells, fibroblasts, endothelial cells, and smooth muscle cells [70]. Functionally, overexpression of CCN1 promoted cell proliferation and protected lung epithelial cells from hyperoxia-induced cell death via the activation of Akt and the downstream pathway [70,82]. Furthermore, the interaction between CCN1 and caveolin-1 (CAV-1) was demonstrated in bronchial epithelial cells [82]. The secretion of CCN1 to the extracellular matrix is controlled by CAV-1, one of the principal structural components of the caveolae [71]. In contrast, by overexpression of CCN1 using adenovirus in vivo, Grazioli et al. observed that CCN1 plays an essential role in the development of ALI by enhancing lung inflammation [40]. Furthermore, the same team proposed that bleomycin-induced CCN1 exacerbates the pathogenesis of ALI [40]. This finding differs from the protective role of CCN1 in lung epithelial cells reported earlier [70,83], suggesting the complex, context-specific, and paradoxical roles of CCN1 in ALI progression. Besides the studies in ALI, a few additional studies have identified CCN1 in association with the pathogenesis of BPD. For instance, CCN1 and CCN2 were dramatically increased in the early stages of injurious ventilation and could serve as an early marker of ALI and VALI [84]. On the other hand, a study showed that exposure to hyperoxia downregulated CCN1 expression in the neonatal rat lungs [34]. Recombinant CCN1 protein treatment exhibited an anti-inflammatory effect and attenuated hyperoxia-induced lung injury in neonatal rats [34], suggesting that CCN1 has potential utility in BPD diagnosis and development of future therapeutics. Although these studies suggest that the dysregulation of CCN1 is tightly associated with ALI and BPD, the development of future therapeutics to target CCN1 in the treatment of these diseases will only become a reality once the exact role of CCN1 and the basic mechanisms by which it promotes these pathologies will be identified.

### 3.2. CCN1 with Chronic Obstructive Pulmonary Disease (COPD)

COPD is a common lung disease that causes irreversible alveoli collapse and an increased risk of developing lung cancer and cardiovascular disease [85,86]. Cigarette smoking (CS) is a major risk factor for developing COPD [85,86]. The gene expression patterns of lung tissues from COPD (GOLD-2) and control smokers (GOLD-0) were compared in clinical research [87]. In this study, the expression of CCN1 was consistently higher in GOLD-2 relative to GOLD-0 cohorts as detected using both the serial analysis of gene expression protocols and tissue microarray analysis. Furthermore, elevated CCN1 was stained in the alveolar epithelial cells, small airway epithelial cells, stromal cells, and inflammatory cells in lung tissues from COPD patients [72]. Additional evidence from a recent report showed increased mRNA expression levels of CCN1 in COPD patients compared to non-smokers [88]. To further explore the mechanism, researchers focused on the regulation of CCN1 in lung epithelium exposed to CS demonstrated that CS increased CCN1 expression and secretion in lung epithelial cells via the induction of reactive oxygen spices (ROS) and endoplasmic reticulum (ER) stress [72]. Hypersecretion of CCN1 resulted in the activation of the Wnt signaling pathway and enhanced release of IL-8, which triggers CS-associated lung inflammation [72]. Besides inflammation, CCN1 also regulates lung epithelial cell survival. CCN1 cleavage was detected in CS exposed lung epithelial cells and lung tissues [44]. Cleaved CCN1 (cCCN1) was released from exosome-shuttled full-length CCN1 (flCCN1) in response to CS treatment [44]. Unlike flCCN1, cCCN1 promoted protease and matrix metalloproteinase (MMP)-1 production after CS exposure and contributed to the epithelial cell death [44].

### 3.3. CCN1 with Pulmonary Fibrosis

Pulmonary fibrosis is a chronic lung disease and leads to severe damage to lung structure and function [89]. Although many studies were conducted to uncover the role of CCN1 in fibrosis, there has been considerable scientific debate going on for decades. Profibrotic effects of CCN1 were observed in different in vivo injury models, including kidney and lung [5]. Kurundkar et al. showed that CCN1 was upregulated in lung tissues of idiopathic pulmonary fibrosis patients [81]. Elevated CCN1 in response to lung injury induced profibrotic gene expression via the TGF-β1/SMAD3 pathway leading to lung fibrosis [81]. A recent study was performed by Kulkarni et al. based on human clinical data, who also demonstrated the potential role of CCN1 in mediating pro-fibrotic effects via enhanced TGF-β1/SMAD signaling [90].

On the other hand, evidence has been provided indicating CCN1 exerting an antifibrotic role in tissue injury repair by promoting fibroblast senescence and apoptosis [9]. Treatment with CCN1 promoted myofibroblast senescence and decreased the amount of collagen deposited during wound healing [9]. Mechanistically, CCN1 triggered fibroblast senescence and the expression of antifibrotic genes by inducing DNA damage response and p53 activation in addition to the generation of reactive oxygen species (ROS) [9]. Intriguingly, CCN1 showed no difference between senescent lung fibroblasts and control groups from patients [9]. More studies are required to address this discrepancy and identify the exact role of CCN1 in progressive lung fibrosis in vivo.

### 3.4. CCN1 with Pulmonary Hypertension (PH)

Pulmonary hypertension (PH) is characterized by increased blood pressure within the pulmonary arteries [91,92]. PH can lead to heart failure and death and has no cure, which makes it a devastating disease [91,92]. Using the hypoxia-induced PH model, we found upregulated CCN1 in the pulmonary vessels and lung parenchyma [93]. Functionally, bioactive recombinant CCN1 significantly suppressed hypoxia-induced contraction in human pulmonary artery smooth muscle cells (PASMCs) [93]. Consistently, administration of bioactive CCN1 significantly decreased right ventricular pressure, suggesting a protective role in PH [93]. A study evaluated the expression of CCN1 in patients with pulmonary arterial hypertension (PAH), which belongs to the first type of PH [94]. Supportively, a significant increase in plasma CCN1 was shown in PAH patients [95]. Furthermore, CCN1 was found to promote PASMC proliferation and contributed to the pathogenesis of PAH [94]. Future investigations will likely provide a better understanding of CCN1 on the pathogenesis of PH.

### 3.5. CCN1 with Lung Infection

Gas exchange takes place in the lungs, where it provides an open environment and is most frequently targeted by pathogens. Lung infection can be caused by bacteria such as *Mycobacterium tuberculosis*, *Klebsiella pneumonia*, and *Streptococcus pneumoniae*, or viruses such as influenza virus and respiratory syncytial virus [96]. Studies revealed that CCN1 regulates innate immune response in the lung during bacterial and viral-induced lung infection. A study from the cecal ligation and puncture rodent model provided the first evidence for dysregulation of CCN1 and CCN2 in the early stages of sepsis in the lung [97]. The pulmonary CCN1 and CCN2 mRNAs displayed 3.3- and 1.4-fold induction, respectively [97]. Lipopolysaccharide (LPS) is the major component of the outer membrane of G-bacteria [98]. The mouse model of LPS-induced lung inflammation is an established model to mimic the cascade of lung infection responses in humans [98]. Previous studies have shown different observations of CCN1 expression in LPS-induced lung inflammation [45,70,77,83]. The inconsistent data could come from the dose, time, or method of LPS administration. The work presented by Cohen et al. showed *Staphylococcus aureus*-secreted virulence factor alpha toxin significantly inhibits CCN1 expression in alveolar macrophage (AM) and leads to the reduced ability of AM to clear neutrophils during *S. aureus* pneumonia [99]. A previous study also demonstrated that Gram-negative bacterial infection causes the hypersecretion of CCN1 [71].

Another study showed that the bacterial DNA and synthetic CpG oligonucleotides promote the secretion of anti-inflammatory CCN1 from lung epithelial cells [37]. The impact of nontypeable *Haemophilus influenzae* (NTHi) infection on the expressions of the CCN family was also evaluated, and it was found that the expressions of both CCN1 and CCN2 isoforms were rapidly induced by NTHi infection in lung tissue [37]. Moreover, influenza A virus infection also upregulates CCN1 and CCN2 [37]. Most recently, Jun reported that CCN1 serves as an opsonin that mediates bacterial clearance via directly binding to TLR2 and TLR4 in response to *Staphylococcus aureus* or *Pseudomonas aeruginosa* infection [65]. In macrophages, CCN1 promotes both phagocytosis and ROS generation, thus enhancing bacterial killing [65]. Mechanistically, they demonstrated that CCN1 activates phagocytosis by engagement of integrin αvβ3 in phagocytes. On the other hand, CCN1 stimulates ROS generation via the activation of Rac1 and NOX2 [65]. Taken together, these in vivo investigations suggested the critical role of CCN1 in the pathological processes of bacterial and viral pneumonia.

### 3.6. CCN1 with Lung Cancer 

Cancer is an inevitable topic when discussing potential novel therapy. From the data of estimated cancer cases versus death in the U.S. in 2010, a total number of approximately 1.5 million were confirmed and 570,000 patients died, which is about a 37% death rate [100,101]. In the updated statistical data in 2019, about 1.7 million patients were diagnosed with various cancer, and 606,000 of them have sadly to be reported as having died [101]. Among the different cancer types, lung cancer is one of the most deadly cancers in the world. In 2010, approximately 222,000 patients were confirmed to have lung cancer, and the death rate was 70%. In the year 2019, although cancer mortality rates have been decreasing in many countries, the death rate of lung cancer is still high (62%, 142,000 out of 228,000), as indicated when comparing the death rate of lung cancer to other primary cancers, such as digestive system cancers, breast cancers, and genital system cancers, whose death rate was around 49%, 19%, and 20% in the year 2010, and 50%, 15%, and 22% in the year 2019, respectively [101]. The statistical data show us the high motility rate of lung cancer compared to other cancers, so it is believed that an improvement in the diagnosis and therapy is very important for patients with lung cancer. A brief summarization of various lung cancer research in association with the CCN1 is described in this section. 

Numerous studies have shown that the CCN1 might serve as a marker for lung cancers. Tong et al. (2004) reported that the mRNA level of CCN1 was decreased in 74 out of 94 lung tumor samples screened, comparing to the normal lung samples [102], and within various lung cancers, the non-small cell lung cancer (NSCLC) attracted most of the attention from researchers, since more than 80–85% of the lung tumors lie within this category [103]. CCN1 protein has been demonstrated by many researchers to participate in the development of NSCLC. A group of researchers has shown that the CCN1 may serve as a tumor suppressor in NSCLC. They have found that overexpression of CCN1 in NSCLC cell lines NCL-H520 and H460 lead to a decrease of colony formation, as well as a remarkable reduction of proliferation compared with the cells stably transfected with empty vector [21]. Consistent with these in vitro findings, both of the CCN1-stably transfected NSCLC cell lines developed smaller tumors than those formed by the control ones in nude mice [21]. In their follow-up study, they demonstrated that p53 plays a pivotal role in CCN1-induced growth arrest using p53 mutant NSCLC cell lines. Furthermore, they fully explored the pathway that CCN1 is involved in and found that overexpressed CCN1 increases c-myc via the activation of the beta-catenin/TCF4 complex. Thus, the upregulated c-myc contributes to p53 signaling pathway activation, which is known to play a central role in cell growth inhibition [104]. Inconsistently, CCN1 demonstrated potential as an oncogene. In a therapeutic study, Li et al. evaluated the therapeutic effects of neutralizing antibody against CCN1 protein in NSCLC cells and mice with NSCLC [105]. They found that CCN1 neutralizing antibody suppressed the epithelial–mesenchymal transition (EMT) signaling pathway in NSCLC cells and reduced NSCLC cell viability. An in vivo study also showed the inhibition of tumor growth and metastasis following anti-CCN1 treatment [105]. 

Besides the proliferation, previous studies suggested that CCN1 may be involved in tumor cell migration and invasion. The authors found that tumor cell-secreted CCN1 can facilitate cell migration via its direct interaction with the αVβ5 integrin in the tested lung carcinoma cell H1155 and H2122 [106]. Lung cancer caused by metastasis is also found to an important topic to investigate. Sabile et al. (2011) showed that the CCN1 is involved in osteosarcoma (SAOS-2 cell) metastasis to the lung in the animal model used in their study. The mice intratibially injected with the CCN1 overexpressing SAOS-2 cell showed more aggressive tumor growth and lung metastases accompanied by a short survival period [107]. A similar study by Fromigue et al. reported that the silencing of CCN1 could promote the apoptosis of the cancer cells and reduce the migration ability of the osteosarcoma cell line [108]. CCN1 was found to be involved in the vascularization and dissemination of by Habel et al. using a lentiviral transduction-induced CCN11 silencing system, the authors concluded that CCN1 silencing could reduce the tumor vasculature and limit the osteosarcoma metastatic capacity, indicating CCN1 is a critical contributor to the tumor vascularization [109].

Another study from Huang et al. revealed that CCN1 plays an essential role in breast cancer lung metastasis. By injecting the cancer cell to the mice model, the authors observed that constitutive CCN1 silencing decreased breast cancer lung metastasis. However, CCN1 silencing using a doxycycline-inducible shRNA system 24 h after cancer cell injection did not influence the lung metastasis [110]. Their study also indicated the CCN1-β1 integrin–AMPKα axis may play a role in breast cancer metastasis to the lung.

Studies indicated the therapeutic effects of some anticancer drugs were mediated by CCN1 expression. In a study led by Jin et al., they evaluated a synthetic compound called norcantharidin (NCTD) in NSCLC cells. The combined treatment of cisplatin and NCTD significantly inhibited Yes-associated protein 1 (YAP)-induced anti-apoptotic effects, EMT via the downregulation of connective tissue growth factor (CTGF), and CCN1 [111]. Another study on NSCLC reported that dexamethasone, which is widely approved for combination therapy in patients with NSCLC, reduced TGF-β1-induced CCN1 expression in NSCLC cells [112]. The result also supported the essential role of CCN1 in NSCLC migration and invasion via the EMT process.

The regulation and function of CCN1 in lung cancer, especially in NSCLC, is still unclear and controversial. Based on the current studies, it will be difficult for researchers to summarize their studies and develop a well-described mechanism for patients. The paradoxical effect of the CCN1 protein has been reported by many researchers, in which it can serve as both tumor suppressor and oncogene. Further investigation is expected to address the inconsistent observations before CCN1 could serve as a diagnostic mark or drug target in NSCLC. Once researchers have come up with the “ideal CCN1 level” on healthy humans with future studies, lung cancers like the NSCLC or those caused by the other cancer metastasis can be screened on time. Therefore, necessary cancer screening and tests can be applied to the patients in the early stage to maximize their chance of survival. 

## 4. Conclusions and Future Perspectives

In our review, information from studies on the CCN1 proteins in lung diseases was gathered and analyzed for its relevance in various lung pathologies. Our literature analyses strongly suggest that the dysregulation of CCN1 occurs in several pulmonary diseases. Furthermore, this review summarizes the current understanding of the signaling pathways in pulmonary diseases. All the above-mentioned signaling pathways are depicted in Figure 1. However, the mechanisms involved in the regulation of CCN1 in these diseases, as well as the mechanisms by which it transduces signals to induce and/or exacerbate lung pathologies are largely unknown. The paradoxical effects of CCN1 have been reported in lung diseases, such as ALI, pulmonary fibrosis, and lung infection. One explanation is CCN1 has different cell surface receptors in a cell type-dependent manner, which leads to various functions. To think further, post-transcriptional modifications of CCN1 may contribute to the different observations. For example, it has been validated that post-transcriptional pre-mRNA processing could be involved in the regulation of CCN1 expression in breast cancer [113]. Additionally, many microRNAs have been reported to act as post-transcriptional regulators of CCN1 [88,114]. On the other hand, the post-translational modification of CCN1 protein was identified [114]. In this report, the O-fucosylation of CCN1 at Thr^242^ was demonstrated using mass spectrometry. Functionally, the O-fucosylation of CCN1 regulates its trafficking from the Golgi apparatus to the extracellular matrix [115]. Moreover, CCN1 bioactivity could be altered by growth factors, cytokines, and enzymes in the cellular microenvironment [5,44], which might lead to the complexity of its biological function. Based on the results discussed above, post-transcriptional/translational modifications and bioactivity of CCN1 should be taken into consideration in future studies. In conclusion, our knowledge of the mechanisms of CCN1 gene regulation and its role in lung pathologies conflicts between various research groups, which could be due to the different models, species, doses of reagents used, and/or stage at which the data was analyzed in addition to CCN1′s context-specific effects. In particular, the regulation of CCN1 may vary among cell types. Future investigations are warranted to address these questions and resolve the discrepancies.

## Figures and Tables

**Table 1:** Tissue and cell distribution of CCN1.

Tissues/Organs	Cell Types	References
Lung	Epithelial cell	[44,45]
Skin	Fibroblasts	[46,47]
Nervous system	Neurons	[48]
Liver	Hepatic stellate cells, fibroblasts	[41,49]
Kidney	HUVEC, podocytes	[43,50]
Bone	Osteoblast cell	[51]
Heart	Endocardial cells, mesenchymal cells	[42]
Muscle	Muscle progenitor cells	[52]
Intestine	Intestinal epithelial cell	[24]
Lymph node	Cancer cells	[53]
Pancreas	Epithelial	[54]
Spleen	Spleen dendritic cells	[50,55]
Stomach	Epithelial	[56]
Eye	Chorioretinal vessel endothelial cells	[57]

**Table 2:** CCN1 biological functions and receptors.

Cell Types Involved	Biological Functions	Membrane Receptors	References
Fibroblast, smooth muscle cells	Cell adhesion	Aα6β1 integrin and HSPGs	[4,57]
Fibroblast	Apoptosis	Aα6β1 integrin	[61]
Endothelial cell	Cell survival	αVβ3 integrin	[57,62]
Astrocytoma cells	Proliferation	α5, α6 and β1 integrins	[66]
Osteoblast	Differentiation	αVβ3 integrin	[51]
Fibroblast	Migration	αVβ5 integrin	[4,67]
Endothelial cell	Angiogenesis	αVβ3 integrin	[31,55]
Macrophages	Inflammation and bacterial clearance	αMβ2 integrin, αVβ3 integrins and TLR2/4	[6,65,68]
Endothelial cell	Survival	αVβ3 integrin	[4,69]
Fibroblasts	Senescence	α6β1 integrin and HSPGs	[4,8]
Epithelial cell	Cell death	α7 integrin	[44,70]
Epithelial cell	Innate immune homeostasis	αVβ6 integrin and LRP6	[71,72]

## Abbreviations

CCN1	Cysteine-rich 61, connective tissue growth factor, and nephroblastoma-1
ALI	Acute lung injury
COPD	Chronic obstructive pulmonary disease
PAH	Pulmonary arterial hypertension
IGFBP	Insulin-like growth factor binding protein
vWC	Willebrand factor type C repeat
TSP	Thrombospondin type 1 repeat
CT	Cysteine knot
TGF-β1	Transforming growth factor β1
FGF2	Fibroblast growth factor
VEGF	Vascular endothelial growth factor
GH	Growth hormone
PDGF	Platelet-derived growth factor
BPD	Bronchopulmonary dysplasia
ARDS	Acute respiratory distress syndrome
VILI	Ventilator-induced lung injury
ROS	Reactive oxygen species
ER	Endoplasmic reticulum
PASMCs	Pulmonary artery smooth muscle cells
LPS	Lipopolysaccharide
NTHi	Nontypeable *Haemophilus influenzae*
NSCLC	Non-small cell lung cancer
EMT	Epithelial–mesenchymal transition
NCTD	Norcantharidin
YAP	Yes-associated protein 1
CTGF	Connective tissue growth factor
